# Factors associated with peptic ulcer perforations in Uganda: a multi-hospital cross-sectional study

**DOI:** 10.1186/s12876-024-03285-w

**Published:** 2024-06-17

**Authors:** Isaac Edyedu, Francis Xaviour Okedi, Joshua Muhumuza, Daniel Asiimwe, Goretty Laker, Herman Lule

**Affiliations:** 1 Department of Surgery, Faculty of Clinical Medicine and Dentistry, Kampala International University Western Campus, PO. Box 70, Ishaka-Bushenyi, Uganda; 2 Faculty of Clinical Medicine and Dentistry, Department of pediatrics and child health, Kampala International University Western Campus, Ishaka-Bushenyi, Uganda; 3 Injury Epidemiology and Prevention Research Group, Division of Clinical Neuroscience, University of Turku, Turku, Finland

**Keywords:** Peptic ulcer disease perforation, Patterns, Low income country

## Abstract

**Introduction:**

Perforated peptic ulcer is the worst complication of peptic ulcer disease whose burden is disproportionately higher in low-income settings. However, there is paucity of published data on the patterns of perforated peptic ulcer in the region. The aim of this study was to determine the factors associated with anatomical patterns of peptic ulcer perforation, as well as the clinical, socio-demographic, and anatomical patterns among patients in Uganda.

**Methods:**

This was a cross sectional study that enrolled 81 consecutive patients with perforated peptic ulcers. Using a structured pretested questionnaire the social demographic and clinical characteristics were obtained. At surgery, the patterns of the perforations were determined. Logistic regression was done in SPSS version 22 to determine the factors associated with the anatomical patterns.

**Results:**

Perforated peptic ulcer disease was more prevalent among males (79.5%), peasants (56.8%) and those from rural areas (65.4%). Majority of study participants were of blood group O (43.2%). Gastric perforations were more common (74.1%). Majority of the perforations were found anteriorly (81.5%). Being a casual laborer was independently associated with lower odds of having a gastric perforation compared to being a peasant farmer (*P* < 0.05).

**Conclusion:**

Public health campaigns aimed at prevention of peptic ulcer perforations should prioritize the males, peasants and those living in rural areas. When a patient in our setting is suspected to have a peptic ulcer perforation, the anterior part of the stomach should be considered as the most likely site involved more so in peasant farmers.

**Supplementary Information:**

The online version contains supplementary material available at 10.1186/s12876-024-03285-w.

## Background

Perforated peptic ulcer is a complication of peptic ulcer disease (PUD) where the ulcer penetrates through the walls of the stomach and duodenum resulting into leakage of digestive juices and food into the abdominal cavity [1]. Globally, over 4,000,000 individuals are affected by peptic ulcer disease annually, with a life time risk of perforation of 5% [1]. Whereas the global incidence of peptic ulcer perforation is reportedly low at 0.19-3% (1–2); its resulting mortality and morbidity is high; up to 30% and 50% respectively (3–4); despite advances in endoscopic diagnostics, *Helicobacter pylori* (*H. pylori*) eradication treatment and use of antacids.

According to Kumar et al. [5], gastro-duodenal perforations account for up to 42% of all gastro-intestinal tract perforations. While the incidence of peptic ulcer perforations is decreasing in developed countries [6], higher rates are still seen in Asia and Africa due to the high burden of *H. pylori* infection caused by poor hygienic practices [6]. *H. pylori* prevalence remains high (90%) in developing countries with higher rates among blood group type O [1], yet *H. pylori* plays a central role in the development of perforated PUD.

Although gastric perforations were previously believed to be more prevalent in developing countries as opposed to duodenal perforations [7], there seems to be a rapid epidemiological transition with developing countries becoming more affluent [3]. Though Uganda has a high burden of PUD [8], existing studies in Uganda, have mostly focused on peptic ulcer disease treatment results and have employed retrospective methodologies with small sample sizes [9]. Therefore, the aim of this study was to determine the factors associated with anatomical patterns of peptic ulcer perforation, as well as the clinical, socio-demographic, and anatomical patterns among patients in Uganda. This knowledge on anatomical patterns of peptic ulcer perforations and associated factors would provide timely information for surgeons to predict the most likely anatomical site of perforation, guiding the operating surgeon to the most likely site and minimizing the operating time. Knowledge of sociodemographic and clinical patterns would help guide focused prevention initiatives.

## Materials and methods

### Study design

This was a hospital-based cross-sectional study on patients with perforated peptic ulcers managed at 6 hospitals in the different regions of Uganda from November 2021 to February 2022.

### Study setting

The study was conducted in the surgical departments of Kampala International University Teaching Hospital (KIU-TH), Fortportal Regional Referral Hospital (FRRH), Hoima Regional Referral Hospital (HRRH), Mubende Regional Referral Hospital (MRRH), Jinja Regional Referral Hospital (JRRH) and Kiryandongo General Hospital (KGH). All these hospitals provide specialized general surgery services including management of patients with acute perforated peptic ulcer disease and they are located in the different regions of Uganda which made the participants representative of the entire country. The surgery teams in these hospitals compromised of general surgeons, surgery residents, intern doctors, anesthesiology staff and nurses.

### Sample size determination and sampling

The sample size was determined by Kish Leslie [10] formula.

$$N=\frac{{Z}^{2}P(1-P)}{{\delta }^{2}}$$

According to a study by Chung & Shelat (1) where the prevalence of perforated peptic ulcer was 5%, *P* = 0.05, Z = 1.96, δ  = 0.05 at 95% level of confidence. On substituting N = 73. On adding 10% to cater for non-responders, the sample size required was 81 participants. The study participants were sampled using consecutive recruitment of all eligible participants until the required sample size was realized.

### Inclusion and exclusion criteria

All patients with gastro-duodenal perforations were included irrespective of age. Patients with traumatic perforations and those in whom the cause was found to be cancer, were excluded.

### Data collection procedure

The respondents who met the inclusion criteria were sensitized about the study and consented post-operatively. The pretested questionnaires were administered by the research assistants and completed post-operatively to document the findings. The variables documented included socio-demographic and clinical characteristics such as age, sex, history of epigastric pain, prior treatment with non-steroidal anti-inflammatory drugs (NSAIDs), alcohol consumption, fasting status, history of cigarette smoking, HIV status, *H-pylori* immunoglobulin G/M status and anatomical site perforated. Laboratory and theatre reports were used as a primary data source to feed the questionnaire for details about clinical characteristics. Patterns of PUD perforation were determined during operation whereas blood group types were determined by tile (slide) and rapid spin (tube) methods. Licensed consultant General surgeons carried out all the operations under observership of surgery residents. The presence of H.pylori was tested using enzyme-linked immunosorbent assay. The sites of the perforations were classified into gastric perforations as an area involving the body, greater curvature, lesser curvature, pre-pyloric, and pyloric, whereas any post-pyloric perforations were classified as duodenal, subdivided as involving the superior, descending, inferior, and ascending parts, also known as 1st, 2nd, 3rd, and 4th parts respectively. For every participant, a tissue sample was taken for histopathology to rule out presence of malignancy. Patients found to have malignancy were excluded.

### Study variables

The dependent variable was the anatomical pattern of the PUD perforation. The independent variables included social-demographic factors and clinical factors.

### Quality control and analysis

The recruited research assistants were trained on how to use the data tool. The principal investigator or his assistant cross checked the data collection tools daily to ensure completeness of the items. Data quality was assured by recruiting only those who met the inclusion criteria. The data tool was completed on the day after surgery for patients who were in the ward and ICU. During administration of the questionnaire, the research assistant used a translated version of the questionnaire to the patients who don’t understand English to ensure consistency of information.

Data was entered in excel, cleaned and exported to SPSS version 22. Percentages and counts of individuals with peptic ulcer perforations were computed for each sociodemographic variable. Frequencies and percentages were computed for each anatomical pattern of perforation. Bivariate and multivariable back ward binary logistic regression was performed to determine the factors associated with gastric or duodenal anatomical patterns.

## Results

Of the 81 patients that had peptic ulcer perforations, majority were males 64(79.0%). Only a few were aged above 50 years 23(28.4%). More than half were peasants 46(56.8%). Majority reported a history of alcohol consumption 44(54.3%). Majority reported previous NSAID use 58(71.6%) and had a positive IgM for H-Pyroli 56(69.1%). Epigastric pain was the commonest symptom at presentation 76(93.8%). The details of participant characteristics are shown in Table 1.

**Table 1:** Socio-demographic and clinical characteristics of patients with perforated peptic ulcer

Characteristic	Frequency	Percentage
**Age**		
1–19	10	12.3
20–29	15	18.5
30–39	20	24.7
40–49	13	16.0
50 and above	23	28.4
**Sex**		
Male	64	79.0
Female	17	21.0
**Occupation**		
Peasant	46	56.8
casual laborer	18	22.2
Student	1	1.2
Business	7	8.6
formal employment	9	11.1
**Estimated Annual income**		
below 1 million	50	61.7
1–5 million	25	30.9
above 5 million	6	7.4
**Residence**		
Urban	28	34.6
Rural	53	65.4
**History of epigastric pain**		
No	5	6.2
Yes	76	93.8
**History of Alcohol consumption**		
No	37	45.7
Yes	44	54.3
**History of smoking**		
No	55	67.9
Yes	26	32.1
**HIV status**		
Negative	72	88.9
Positive	9	11.1
**History of NSAID use**		
No	23	28.4
Yes	58	71.6
**H. pylori IGM status**		
Negative	25	30.9
Positive	56	69.1
**ABO blood group**		
1(A)	19	23.5
2(B)	14	17.3
3(AB)	13	16.0
4(O)	35	43.2

Gastric perforations were more common 60(74.1%) than duodenal perforations 21(25.9%) and mainly occurred anteriorly in both cases 66(81.5%). The commonest site for gastric perforation was the lesser curvature 26(43.3%) followed by the pre-pyloric region 21(35.0%) whereas duodenal perforations mainly affected the first part 18(85.7%) (Table 2).

**Table 2:** Anatomical patterns of peptic ulcer perforations among the study participants

Location	Frequency	Percentage in Specific site
**Gastric (** *N* ** = 60)**		
Fandus	4	6.7
Body	5	8.3
Lesser curvature	26	43.3
Greater curvature	4	6.7
Pre pyloric	21	35.0
**Duodenal (** *N* ** = 21)**		
1st part	18	85.7
2nd part	3	14.3
**Antero/posterior (** *N* ** = 81)**		
Anterior	66	81.5
Posterior	14	17.3
Anterior & Posterior (2)	1	1.2

In bivariate analysis, being a casual laborer was associated with lower odds of having a gastric ulcer perforation compared to being a peasant (OR = 0.122, CI = 0.033–0.452, *P* = 0.002), being a business person was also associated with lower odds of gastric perforation (OR = 0.163, CI = 0.028–0.947, *P* = 0.043) and patients above 50 years had 7 times more odds of having a gastric ulcer perforation compared to those aged 1–19 years (OR = 7.000, CI = 1.021–47.969, *P* = 0.048). In multivariate analysis, being a casual laborer had reduced odds of having a gastric perforation compared to being a peasant farmer (AOR = 0.125, CI = 0.026–0.610, *P* = 0.010) (Table 3). The details of all the variables assessed in the bivariate and multivariate are shown in supplementary file 1.

**Table 3:** Bivariate and multivariate analysis of the factors associated with having a gastric perforation

		Bivariate analysis		Multivariate analysis	
Variable	**Gastric site ** *n* **(%) ** *N* ** = 60**	**COR (95%CI)**	*p-value*	**AOR (95%CI)**	*p-value*
Age					
1–19	6(10.0%)	Ref			
20–29	9(15.0%)	1.000(0.195–5.121)	1.000	0.393(0.029–5.359)	0.483
30–39	13(21.7%)	1.238(0.259–5.913)	0.789	0.634(0.052–7.739)	0.721
40–49	11(18.3%)	3.667(0.513–26.224)	0.196	2.883(0.174–47.853)	0.460
≥ 50	21(35.0%)	**7.000(1.021–47.969)**	**0.048**	2.107(0.155–28.664)	0.576
Occupation					
Peasant	41(68.3%)	Ref			
casual labor	9(15.0%)	**0.122(0.033–0.452)**	**0.002**	**0.125(0.026–0.610)**	**0.010**
Business	4(6.7%)	**0.163(0.028–0.947)**	**0.043**	0.160(0.017-1.500)	0.109
formal employment	6(10.0%)	0.244(0.046–1.293)	0.097	0.473(0.063–3.559)	0.467

## Discussion

In this study, majority of study participants were aged above 50 years and mainly peasants of rural residence with an annual earning of less than one million. Our findings are comparable to those of Katagiri et al. [11]. , and Huang et al. [12]. , in which low social economic status and old age were associated with peptic ulcer perforation. Low socioeconomic status is associated with overcrowding, poor hygiene and high H.Pylori infection. Lobankov [13] and Søreide et al. [14]. , have argued that peptic ulcer perforation is a phenomenon of old age because of concomitant diseases, multiple therapy for the concomitant diseases and reduction in the mucosal protective barrier as a result of aging. In contrast, Chalya et al. [15]. , in Tanzanian noted that the most affected age group was 21–30 years, however their study participants were mainly immunosuppressed with HIV yet in our study only 11.1% of the study participants had HIV, which could explain the difference.

Further, we established that overall, males (79.0%) were over represented. Our findings are in keepings with Gona et al. [16] who documented that 90% of patients who underwent surgery for a perforated peptic ulcer were males. Scholars attribute male over representation to high alcohol consumption rates and cigarette smoking [15]. Researchers in Ethiopia found that men were more likely to smoke, drink alcohol and use NSAIDS but what drives this behavior other than psychosocial stress is poorly documented [17]. In our study, the majority of study participants reported history of alcohol consumption (54.3%) and previous NSAID use (71.6%) but only 32.1% smoked cigarette. These factors have been associated with peptic ulcer perforation in previous studies [18].

Furthermore, the highest prevalence of peptic perforations (43.4%) was amongst individuals with blood group O (43.2%) similar to that reported in Iraq patients (57.5%) [19]. The existing studies indicate mixed findings where some scholars document increased prevalence of gastric ulcers in blood group O [20] whereas others report increased prevalence in blood group B [21]. Some academicians have correlated these differences to biological behavior [22] and susceptibility to H. Pylori [23] which the present study has not resolved. Although majority of our study participants had a positive IgM for H-Pylori (69.1%) this was within the range of 50–80% prevalence of H-pylori infection among perforated peptic ulcer as reported in literature. Our study did not find any statistically significant association between H-Pylori IgM sero positivity and anatomical patterns of peptic ulcer perforation nor was an association found between ABO blood group and the anatomical patterns.

Our findings showed that gastric perforations were more common than duodenal perforations. Our study concurred with findings in Ghana [24] and Nigeria [25]. However, this was in contrast to findings of Bhardwaj [26], Khan [27] and a Rwandese study by Rickard [28], in which duodenal perforations were over represented. In addition, our findings were incongruent to those from Tanzania [15], Kenya [29] and Southern Sudan [30] where the ratio of gastric to duodenal perforations were 1:12.7, 1:11.5 and 1:25 respectively. The increasing occurrence of gastric ulcers in low income countries could be as a result of H-pylori infection [31] and might depict an epidemiological disease transition in developing countries.

Further, we found that peptic ulcer perforations mainly occurred anteriorly in all cases (79.2%) probably because of no protective structures anteriorly compared to the posterior wall. Posterior perforations present with bleeding and abscess formation as opposed to acute abdomen in the anterior perforations. The commonest site for gastric perforation being the lesser curvature (43.3%) followed by the pre-pyloric region (35.0%). These are sites for benign disease compared to sites like antrum, greater curvature and fundus where malignant ulcers tends to perforate whereas duodenal perforations mainly affected the first part (85.7%) than the second part probably because its retroperitoneal with additional protective support [32]. Our results are similar to those of Zittel [32] and Bertleff [33] in which peptic ulcer perforations mostly occurred on anterior wall of the duodenum (60%), antrum (20%) and lesser-curvature of the stomach (20%).

Though being older than 50 years was associated with increased risk of gastric perforation, only being a casual laborer was independently associated with lower odds of gastric ulcer perforations compared to being a peasant farmer. This could be because of physical activity and moderate leisure time among these people which seems to protect even the patients with H-pylori infection from getting PUD and its complications. Katagiri et al. [11]. , and Huang et al. [12]. , have reported high occurrence of peptic ulcers perforations in individuals of low socioeconomic status which is associated with poverty, poor hygiene, high H-pylori infection and delay in seeking medical treatment.

### Study limitations

The research was designed to be cross-sectional, as a result, it was not possible to draw any conclusions about the cause-and-effect relationships. More so, we did not collect data on the patient outcomes.

## Conclusion

This study established that perforated peptic ulcer disease was more prevalent amongst male peasants of rural residence. The gastric perforations were more common than duodenal perforations but both perforations mainly occurred anteriorly. Being a casual laborer was independently associated with lower odds of gastric ulcer perforation compared to being peasant farmer. This study has shed light on most common clinical patterns of peptic ulcer perforation in our local setting. A background knowledge of these patterns amongst surgeons could enhance making timely intraoperative decisions for these patients.

### Recommendations

Public health campaigns aimed at prevention of peptic ulcer perforations should prioritize the males, peasants and those living in rural areas. When a patient in a low resource setting is suspected to have a peptic ulcer perforation, the anterior part of the stomach should be considered as the most likely site involved more so in peasant farmers. Future studies should be long term prospective cohorts to establish causation in addition to reporting outcomes of management.

### Electronic supplementary material

Below is the link to the electronic supplementary material.

Supplementary Material 1

## Data Availability

No datasets were generated or analysed during the current study.

## Abbreviations

PUD: Peptic ulcer disease
HIV: Human immune-deficiency virus
NSAIDs: Non-steroidal anti-inflammatory drugs
