# Supplementary material for: Factors associated with peptic ulcer perforations in Uganda: a multi-hospital cross-sectional study
Source: BMC Gastroenterol. 2024 Jun 17;24:199. doi: 10.1186/s12876-024-03285-w (PMC11181620; doi:10.1186/s12876-024-03285-w)
Supplement: Supplementary file 1 — Supplementary Material 1 [file 12876_2024_3285_MOESM1_ESM.docx]

**Table S1: Bivariate analysis of factors associated with anatomical patterns of peptic ulcer perforation**

| **Characteristic** | **Duodenal perforation, N(%)** | | **Gastric perforation, N (%)** | **Crude OR** | **95% confidence Interval** | | **P-Value** |
| --- | --- | --- | --- | --- | --- | --- | --- |
|  |  |  |  |  | **Lower Limit** | **Upper Limit** |  |
| **Age** | | | | | | | |
| 1-19 | 4 | 6 | | Ref |  |  |  |
|  | 19.0% | 10.0% | |  |  |  |  |
| 20-29 | 6 | 9 | | 1.000 | 0.195 | 5.121 | 1.000 |
|  | 28.6% | 15.0% | |  |  |  |  |
| 30-39 | 7 | 13 | | 1.238 | 0.259 | 5.913 | 0.789 |
|  | 33.3% | 21.7% | |  |  |  |  |
| 40-49 | 2 | 11 | | 3.667 | 0.513 | 26.224 | 0.196 |
|  | 9.5% | 18.3% | |  |  |  |  |
| 50 and above | 2 | 21 | | 7.000 | 1.021 | 47.969 | 0.048 |
|  | 9.5% | 35.0% | |  |  |  |  |
| **Sex** | | | | | | | |
| Male | 17 | 47 | | Ref |  |  |  |
|  | 81.0% | 78.3% | |  |  |  |  |
| Female | 4 | 13 | | 1.176 | 0.337 | 4.105 | 0.800 |
|  | 19.0% | 21.7% | |  |  |  |  |
| **Religion** | | | | | | | |
| Christian | 18 | 49 | | Ref |  |  |  |
|  | 85.7% | 81.7% | |  |  |  |  |
| Muslim | 3 | 11 | | 1.347 | 0.337 | 5.387 | 0.674 |
|  | 14.3% | 18.3% | |  |  |  |  |
| **Education level** | | | | | | | |
| None | 3 | 8 | | Ref |  |  |  |
|  | 14.3% | 13.3% | |  |  |  |  |
| Primary | 8 | 29 | | 1.359 | 0.291 | 6.344 | 0.696 |
|  | 38.1% | 48.3% | |  |  |  |  |
| Secondary | 7 | 18 | | 0.964 | 0.197 | 4.721 | 0.964 |
|  | 33.3% | 30.0% | |  |  |  |  |
| Tertiary | 3 | 5 | | 0.625 | 0.089 | 4.401 | 0.637 |
|  | 14.3% | 8.3% | |  |  |  |  |
| **Occupation** | | | | | | | |
| Peasant | 5 | 41 | | Ref |  |  |  |
|  | 23.8% | 68.3% | |  |  |  |  |
| casual labor | 9 | 9 | | 0.122 | 0.033 | 0.452 | 0.002 |
|  | 42.9% | 15.0% | |  |  |  |  |
| Business | 3 | 4 | | 0.163 | 0.028 | 0.947 | 0.043 |
|  | 14.3% | 6.7% | |  |  |  |  |
| formal employment | 3 | 6 | | 0.244 | 0.046 | 1.293 | 0.097 |
|  | 14.3% | 10.0% | |  |  |  |  |
| **Estimated Annual income** | | | | | | | |
| **Below 1 million** | 14 | 36 | | Ref |  |  |  |
|  | 66.7% | 60.0% | |  |  |  |  |
| **1-5 million** | 6 | 19 | | 1.231 | 0.407 | 3.722 | 0.712 |
|  | 28.6% | 31.7% | |  |  |  |  |
| **above 5 million** | 1 | 5 | | 1.944 | 0.208 | 18.156 | 0.560 |
|  | 4.8% | 8.3% | |  |  |  |  |
| **Residence** | | | | | | | |
| **Urban** | 9 | 19 | | Ref |  |  |  |
|  | 42.9% | 31.7% | |  |  |  |  |
| **Rural** | 12 | 41 | | 1.618 | 0.583 | 4.494 | 0.355 |
|  | 57.1% | 68.3% | |  |  |  |  |
| **History of epigastric pain** | | | | | | | |
| **No** | 3 | 2 | | Ref |  |  |  |
|  | 14.3% | 3.3% | |  |  |  |  |
| **Yes** | 18 | 58 | | 4.833 | 0.748 | 31.226 | 0.098 |
|  | 85.7% | 96.7% | |  |  |  |  |
| **History of Alcohol consumption** | | | | | | | |
| **No** | 12 | 25 | | Ref |  |  |  |
|  | 57.1% | 41.7% | |  |  |  |  |
| **Yes** | 9 | 35 | | 1.867 | 0.683 | 5.100 | 0.224 |
|  | 42.9% | 58.3% | |  |  |  |  |
| **History of smoking** | | | | | | | |
| **No** | 16 | 39 | | Ref |  |  |  |
|  | 76.2% | 65.0% | |  |  |  |  |
| **Yes** | 5 | 21 | | 1.723 | 0.553 | 5.365 | 0.348 |
|  | 23.8% | 35.0% | |  |  |  |  |
| **HIV status** | | | | | | | |
| **Negative** | 18 | 54 | | Ref |  |  |  |
|  | 85.7% | 90.0% | |  |  |  |  |
| **Positive** | 3 | 6 | | 0.667 | 0.151 | 2.943 | 0.593 |
|  | 14.3% | 10.0% | |  |  |  |  |
| **History of NSAID use** | | | | | | | |
| **No** | 4 | 19 | | Ref |  |  |  |
|  | 19.0% | 31.7% | |  |  |  |  |
| **Yes** | 17 | 41 | | 0.508 | 0.150 | 1.715 | 0.275 |
|  | 81.0% | 68.3% | |  |  |  |  |
| **H-Pylori IGM status** | | | | | | | |
| **Negative** | 7 | 18 | | Ref |  |  |  |
|  | 33.3% | 30.0% | |  |  |  |  |
| **Positive** | 14 | 42 | | 1.167 | 0.403 | 3.374 | 0.776 |
|  | 66.7% | 70.0% | |  |  |  |  |
| **ABO blood group** | | | | | | | |
| **1(A)** | 5 | 14 | | 0.700 | 0.188 | 2.607 | 0.595 |
|  | 23.8% | 23.3% | |  |  |  |  |
| **2 (B)** | 4 | 10 | | 0.625 | 0.150 | 2.599 | 0.518 |
|  | 19.0% | 16.7% | |  |  |  |  |
| **3 (AB)** | 5 | 8 | | 0.400 | 0.100 | 1.607 | 0.197 |
|  | 23.8% | 13.3% | |  |  |  |  |
| **4 (O)** | 7 | 28 | | Ref |  |  |  |
|  | 33.3% | 46.7% | |  |  |  |  |

We analyzed results for 81 individuals. Here the one individual who had both duodenal and gastric perforation was recoded to gastric perforation to create the binary outcome for logistic regression. (1-Yes gastric perforation, 0-Duodenal perforation). At bivariate analysis being a casual laborer was associated with lower risk of gastric ulcer perforation compared to being a peasant [OR=0.122(0.033-0.452), P=0.002], being a business person was also associated with lower risk of gastric perforation [OR=0.163(0.028-0.947), P=0.043] and patients above 50 years were 7 times more likely to get gastric ulcer perforations compared to 1- 19 years [OR=7.000(1.021-47.969) P=0.048). People with blood group O were 2.5 times more likely to have gastric perforation compared to these with blood group AB (OR=0.400 (0.100-1.607), P=0.197), and a patient with history of epigastric pain was 4.833 times more likely to have gastric perforation compared to one without epigastric pain (OR=4.833(0.748-31.226), P=0.098), though blood group and history of epigastric pain were slightly above the cut off for the significance as shown in table S1.

**Table S2: Multivariate analysis of factors associated with patterns of peptic ulcer perforation**

| **Characteristic** | **Adjusted OR** | **95% Confidence interval** | | **p-value** |
| --- | --- | --- | --- | --- |
|  |  | **Lower** | **Upper** |  |
| **Occupation** |  |  |  |  |
| Peasant | Ref |  |  |  |
| casual labor | 0.125 | 0.026 | .610 | 0.010 |
| Business | 0.160 | 0.017 | 1.500 | 0.109 |
| formal employment | 0.473 | 0.063 | 3.559 | 0.467 |
| **Blood Group** |  |  |  |  |
| 1(A) | 0.575 | 0.109 | 3.029 | 0.514 |
| 2 (B) | 0.498 | 0.072 | 3.440 | 0.480 |
| 3 (AB) | 0.292 | 0.043 | 1.983 | 0.208 |
| 4 (O) | Ref |  |  |  |
| **Age (Years)** |  |  |  |  |
| 1-19 | Ref |  |  |  |
| 20-29 | 0.393 | 0.029 | 5.359 | 0.483 |
| 30-39 | 0.634 | 0.052 | 7.739 | 0.721 |
| 40-49 | 2.883 | 0.174 | 47.853 | 0.460 |
| 50 and above | 2.107 | 0.155 | 28.664 | 0.576 |
| **Alcohol use** |  |  |  |  |
| No | Ref |  |  |  |
| Yes | 1.731 | 0.422 | 7.105 | 0.446 |
| **Epigastric pain** |  |  |  |  |
| No | Ref |  |  |  |
| Yes | 7.650 | 0.647 | 90.405 | 0.106 |

Variables with P< 0.25 at bivariate were analyzed at multivariate level, this showed that being a casual laborer (p=0.010), was associated with lower risk of gastric ulcer perforations compared to peasant farmers. A Patient with blood group O was 3.42 times more likely to have a gastric perforation compared to one with AB [aOR=0.292 (0.043- 1.983), P=0.208] though the P-value for the blood group was not significant (table S2).
